# Marked host specificity and lack of phylogeographic population structure of *Campylobacter jejuni* in wild birds

**DOI:** 10.1111/mec.12144

**Published:** 2013-01-29

**Authors:** Petra Griekspoor, Frances M Colles, Noel D McCarthy, Philip M Hansbro, Chris Ashhurst-Smith, Björn Olsen, Dennis Hasselquist, Martin C J Maiden, Jonas Waldenström

**Affiliations:** * Centre for Ecology and Evolution in Microbial Model Systems (EEMiS), Linnaeus UniversitySE-391 82, Kalmar, Sweden; † Department of Zoology, University of OxfordSouth Parks Road, Oxford, OX1 3PS, UK; ‡ Centre for Asthma and Respiratory Disease and Hunter Medical Research Institute, University of NewcastleNewcastle, NSW 2300, Australia; § Department of Medical Sciences, Uppsala UniversityUppsala, Sweden; ¶ Department of Biology, Lund UniversityEcology Building, SE-223 62, Lund, Sweden

**Keywords:** disease emergence, epidemiology, host associations, Zoonotic disease

## Abstract

Zoonotic pathogens often infect several animal species, and gene flow among populations infecting different host species may affect the biological traits of the pathogen including host specificity, transmissibility and virulence. The bacterium *Campylobacter jejuni* is a widespread zoonotic multihost pathogen, which frequently causes gastroenteritis in humans. Poultry products are important transmission vehicles to humans, but the bacterium is common in other domestic and wild animals, particularly birds, which are a potential infection source. Population genetic studies of *C. jejuni* have mainly investigated isolates from humans and domestic animals, so to assess *C. jejuni* population structure more broadly and investigate host adaptation, 928 wild bird isolates from Europe and Australia were genotyped by multilocus sequencing and compared to the genotypes recovered from 1366 domestic animal and human isolates. *Campylobacter jejuni* populations from different wild bird species were distinct from each other and from those from domestic animals and humans, and the host species of wild bird was the major determinant of *C. jejuni* genotype, while geographic origin was of little importance. By comparison, *C. jejuni* differentiation was restricted between more phylogenetically diverse farm animals, indicating that domesticated animals may represent a novel niche for *C. jejuni* and thereby driving the evolution of those bacteria as they exploit this niche. Human disease is dominated by isolates from this novel domesticated animal niche.

## Introduction

Many pathogens that infect humans or domestic animals can inhabit multiple animal hosts and environmental reservoirs (Daszak *et al*. 2000; Woolhouse *et al*. 2001). A broad host range of a pathogen can increase the risk of disease emergence in novel hosts (Cleaveland *et al*. 2001). Limited gene flow among distinct pathogen populations inhabiting different hosts may lead to adaptations to specific niches and initiate allopatric speciation (Cohan & Koeppel 2008; Sheppard *et al*. 2008, 2011). The evolutionary implications of a pathogen with a multiple host population structure will depend on the transmission frequencies among and within host species, resulting in a range of outcomes from spillover infections to emerging infectious diseases in the novel host species (Fenton & Pedersen 2005). Understanding and quantifying the genetic structure of populations of pathogenic microorganisms could therefore provide a means to predict the potential for emerging infectious diseases in humans (Cleaveland *et al*. 2001).

*Campylobacter jejuni* is a zoonotic multihost pathogen that has a substantial impact on human health. In humans, *C. jejuni* infections are primarily food-borne, the majority of which are caused by genotypes that are common in food animals, especially poultry (Dingle *et al*. 2001, 2002; Colles *et al*. 2003; Manning *et al*. 2003; Schouls *et al*. 2003; McCarthy *et al*. 2007). Commercially reared poultry are often colonized with *C. jejuni* and can carry high bacterial loads asymptomatically, suggesting commensal adaptations to the avian gut (Humphrey *et al*. 2007). However, although most studies have investigated *C. jejuni* isolates from domestic animals or human patients, the bacterium has a much broader host range. *Campylobacter jejuni* is common in a variety of species of wild birds (Luechtefeld *et al*. 1980; Kapperud & Rosef 1983; Waldenström *et al*. 2002) and has also been identified in several species of pet animals, rodents and insects (Rosef & Kapperud 1983; Sproston *et al*. 2010). In wild birds, *C. jejuni* prevalence rates vary between host species and seem to be linked to diet (Kapperud & Rosef 1983; Broman *et al*. 2002; Waldenström *et al*. 2002). Disease manifestation in wild birds has not been extensively evaluated, but a recent study showed a slight reduction in body mass in European robins, *Erithacus rubecula,* challenged with a *C. jejuni* isolate from another songbird species (Waldenström *et al*. 2010). In humans, the severity of infection varies profoundly from asymptomatic to severe gastrointestinal illness (Dasti *et al*. 2010). Although *C. jejuni* has been mainly studied in relation to its role as a human pathogen, its growth characteristics at different temperatures (i.e. able to grow at 37–42 °C) and frequent isolation from domestic and wild birds suggest that it is primarily an avian bacterium and that wild avian species may function as natural reservoirs for *C. jejuni* (Luechtefeld *et al*. 1980; Hermans *et al*. 2011; Sheppard *et al*. 2011).

To date, *C. jejuni* population genetic analyses have been largely dominated by isolate collections originating from food animals and human campylobacteriosis, although there is increasing interest in the analysis of isolates from environmental sources and wild animals (Broman *et al*. 2002, 2004; French *et al*. 2005, 2009; Colles *et al*. 2008a, 2009; Sheppard *et al*. 2011). The population structure of human and food animal *C. jejuni* isolates is essentially nonclonal and dominated by recombination (Dingle *et al*. 2001; Suerbaum *et al*. 2001; Manning *et al*. 2003), which acts to diminish genetic differentiation among bacterial subpopulations (Broman *et al*. 2002; Dingle & Maiden 2005). Within this population, however, genotyping with multilocus sequence typing (MLST) has identified clonal complexes, groups of related genotypes that are probable to share a common ancestor (Dingle *et al*. 2002). Because isolates from food animals and wild animals often show genetic subdivision, estimates of population genetic parameters obtained from analyses of poultry- or human-dominated data sets can obscure biological and epidemiological properties of *C. jejuni* (Meinersmann 2000).

Earlier studies of wild bird isolates indicate that *C. jejuni* has a high degree of host specificity with little overlap of genotypes between isolation sources including host species (Broman *et al*. 2002; Colles *et al*. 2008a,b, 2009). Recently, Sheppard *et al*. investigated niche segregation in *C. jejuni* and *C. coli* from wild and domestic animal sources in the United Kingdom (Sheppard *et al*. 2011) and found distinct *C. jejuni* genotype assemblages in different groups of birds, strengthening the notion of genotype by host associations. This contrasts to the situation seen among food animal *C. jejuni* genotypes, where host associations are weaker (Sheppard *et al*. 2011), possibly indicating a different ecology in the food animal niche. To further assess the natural ecology of *C. jejuni* and test the predictions from earlier studies at a larger geographical scale, we collected a large set of samples from various wild bird species, farm-related animals and humans, from Europe and Australia and employed MLST (Broman *et al*. 2002; Dingle & Maiden 2005; Mickan *et al*. 2007; Baker *et al*. 2010) to examine the population structure of *C. jejuni* by host and geography. By sampling taxonomically related, but geographically separated species pair, we could specifically address the existence of host-associated *C. jejuni* genotype assemblages among wild bird host and relate this to the ecology of *C. jejuni* in food animals and human disease.

## Materials and methods

### Bacterial isolates

The population genetic analyses were based on 2294 characterized *C. jejuni* isolates. Of these, 928 *C. jejuni* were isolated from wild birds sampled in Sweden (Waldenström *et al*. 2002; Broman *et al*. 2004), the United Kingdom (Colles *et al*. 2008a, 2009) and Australia (Table 1), while the remaining 1366 bacterial MLST genotypes came from published studies on *C. jejuni* in humans in Australia (Kinana *et al*. 2006; Mickan *et al*. 2007) and the United Kingdom (Dingle *et al*. 2008), and in farm animals (Kinana *et al*. 2006; McCarthy *et al*. 2007). The Australian wild bird samples were collected in 2004–2006 in the Newcastle and Melbourne areas. Australian shorebirds were trapped at Stockton Sandpit with funnel walk-in traps or mist nets, and faecal samples were collected from gulls around Newcastle, while faecal samples from passerines were taken at Melbourne Botanic Gardens (Hansbro *et al*. 2010). Details on trapping, sampling and primary isolation procedures for all other samples are given in previously published studies (Broman *et al*. 2002, 2004; Waldenström *et al*. 2002; Colles *et al*. 2008a, 2009). All sampling was performed in accordance with approval from the different national authorities. In analyses of *C. jejuni* population subdivision, we created 17 subsets by combining the source of isolation (i.e. song thrush, silver gull, human) and country of isolation (i.e. Australia, Sweden, United Kingdom; Table 1).

**Table 1:** Host origin, sampling year and sampling site of investigated *Campylobacter jejuni* strains

Host species	Latin name	No. of isolates	Year	Country
Mallard	*Anas platyrhynchos*	85	2002	Sweden
Dunlin	*Calidris alpina*	21	2000	Sweden
		8	2001	Sweden
Black-headed gull	*Larus ridibundus*	48	1999	Sweden
		49	2000	Sweden
Starling	*Sturnus vulgaris*	7	2000	Sweden
		285	2002–2005	UK
Geese		166	2002–2003	UK
Blackbird	*Turdus merula*	32	2000	Sweden
		71	2001	Sweden
		4	2006	Australia
Song thrush	*Turdus philomelos*	84	2000	Sweden
Sharp-tailed sandpiper	*Calidris cuminata*	33	2004	Australia
		1	2005	Australia
		12	2006	Australia
Silver gull	*Larus ovaehollandiae*	10	2004	Australia
		12	2006	Australia
Chicken		7	2000	Senegal
		23	2001	Senegal
		16	2002	Senegal
		217	1982–2003	UK
Cattle		218	1984–2001	UK
Sheep		158	1982–2002	UK
Human		574	2003–2004	UK
		153	1999–2001	Australia
Total		2294		

### Genetic characterization

Fresh overnight cultures were used to make either boilates (one loop-full of cells in 1 mL of ddH_2_O boiled for 8 min) or DNA extractions with commercial kits (Puregene DNA Isolation Kit) for the use as template in PCRs. For the majority of the isolates, the species identity had been confirmed by the use of a multiplex PCR assay for delineation of *C. coli* and *C. jejuni* (Vandamme *et al*. 1997), using conditions validated previously (On & Jordan 2003).

The sequence type (ST) of each isolate was determined using the published *C. jejuni* MLST protocol. Fragments of seven housekeeping genes distributed around the bacterial chromosome were amplified by PCR (Dingle *et al*. 2001). The resulting PCR products were sequenced, and the nucleotide extension reactions products were separated and detected on automated DNA analysers. The sequences were assembled and edited, and allele numbers and STs and clonal complexes were assigned using the internet-based *Campylobacter jejuni* and *Campylobacter coli* MLST database (with accession numbers 21601-22077, http://pubmlst.org/campylobacter/).

### Assessment of association of genotype with host species, time and geography

**Fig. 1:**
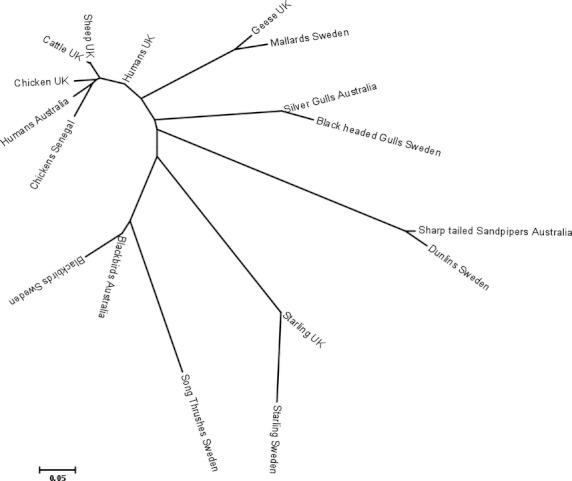
Unrooted neighbour-joining tree displaying the pairwise genetic distances (*F*_ST_ values) between *Campylobacter jejuni* populations from different hosts and geographical areas. The *F*_ST_ values were calculated from nucleotide polymorphisms in the concatenated sequences from seven loci (3709 bp) in the 2294 *C. jejuni* isolates. All differences between sources were significant at *P* < 0.05.

The sequence alignment of the concatenated sequences of all seven loci was exported to the software DnaSP, version 4.10 (Rozas *et al*. 2003), in which subsets (based on host species and geography) were created and nucleotide-based analyses of gene flow and genetic differentiation were assessed. A similar analysis at the level of alleles was conducted with the software Arlequin, version 2.00 (Schneider *et al*. 2000). Resulting *F*_ST_ values were exported as genetic distances and visualized with neighbour-joining trees in mega5 (Kumar *et al*. 2001) (Fig. 1 and S1, Supporting information). Additional analyses of genetic subdivision depending on host species, and of molecular variances (amova), were calculated in Arlequin.

**Fig. 2:**
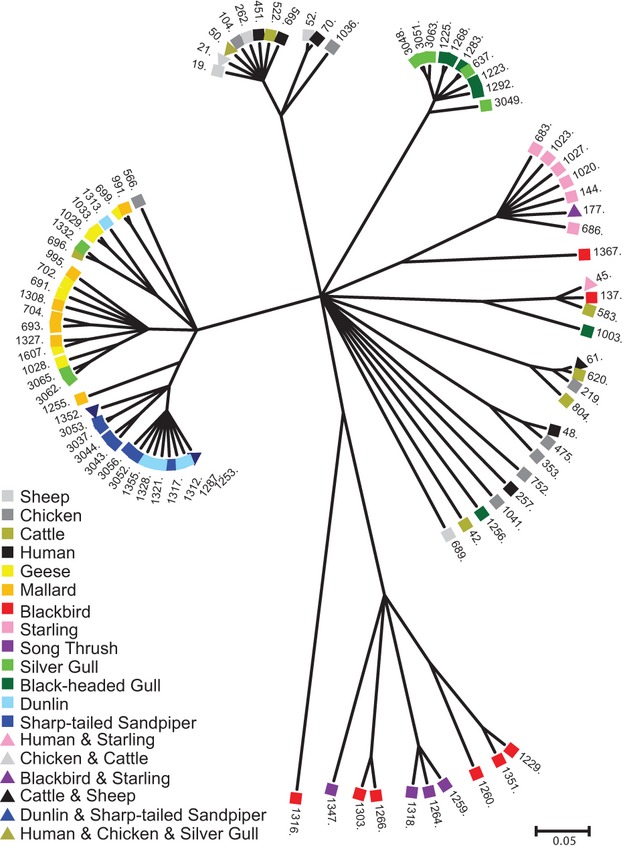
A ClonalFrame genealogy of *Campylobacter jejuni* STs from wild birds, food animals and humans. From each of the 13 host species, 10 random *C. jejuni* isolates were drawn and included in the analysis. The source of isolates is indicated with different colour (see inset in the figure), and STs are given in numbers at the tip of branches.

Phylogenetic relationships were illustrated using two different approaches. In the first analysis, we used the ClonalFrame software to construct phylogenies that incorporate both mutation and recombination (Didelot & Falush 2007). A random set of 10 isolates from each of the 13 host species was drawn and run at default values with 50 000 burn-in iterations and 50 000 further iterations from which each 100th tree was sampled. Four independent runs were conducted, and convergence was estimated with Gelman Rubin statistics (Didelot & Falush 2007). A 75% consensus tree of the combined data from four runs was constructed with ClonalFrame and exported as a Newick tree for display and labelling in mega5 (Fig. 2). In the second analysis, we used the software goeburst (Francisco *et al*. 2009), which uses the same clustering rules as the frequently used eburst (Feil *et al*. 2004) but which provides a global optimal solution, to determine the clonal relationships between STs of the entire data set. The software calculates a minimum spanning tree and identifies predicted founder STs and any single locus variant to those genotypes (Fig. S2, Supporting information).

### Isolate assignment to hosts

**Fig. 3:**
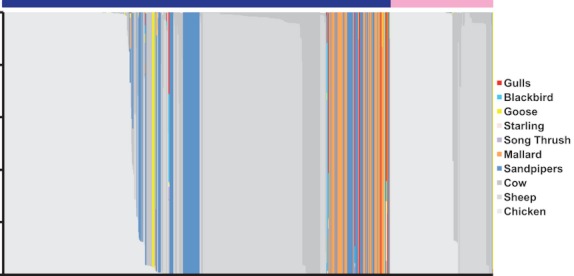
Assignment of human isolates to food animals and wild bird populations. The probabilistic assignment of the *Campylobacter jejuni* host population was based on allele frequencies using the software structure. Each allelic profile is represented by a vertical bar, showing the estimated probability that it comes from each of the source identified. The horizontal coloured bar above the vertical bars indicate whether human isolates were from UK (blue) or from Australia (pink). Food animal related isolates are shown in shades of grey, while the isolates of different wild birds are denoted in colours: silver gull (light green), black-headed gull (dark green), blackbird (red), song thrush (purple), European starling (pink), goose species (yellow), mallards (orange), sharp-tailed sandpiper (dark blue), dunlin (light blue).

Assignment of isolates from humans to food animal and wild bird populations was undertaken using the population genetic assignment software structure (Falush *et al*. 2003). We reduced the number of reference populations by combining closely related *C. jejuni* populations from similar wild bird taxa. Thus, *C. jejuni* from sharp-tailed sandpipers and dunlins were treated as one group and *C. jejuni* from silver gulls and black-headed gulls as another group. The combining of host species harbouring similar *C. jejuni* populations was performed to ensure that reference populations were all of at least size 50. The structure algorithm provides unbiased assignment based on the allele frequency assuming independence between alleles. The no-admixture model was used with default values and 5000 burn-in iterations followed by 10 000 sampled iterations in line with published approaches that have used the assignment of *C. jejuni* isolates to host species (McCarthy *et al*. 2007; Sheppard *et al*. 2009). The probability of origin in each of the reference populations is estimated for each human isolate, with a probability between zero and one inclusive for each possible source population summing to 1 across the possible sources (Fig. 3).

## Results

We detected a broad diversity in the number of *C. jejuni* genotypes obtained from wild birds. Approximately one new ST was obtained for every fourth sequenced isolates with 251 unique STs detected from wild birds (Table S1, Supporting information). However, most of these of genotypes were grouped into 22 clonal complexes comprising related genotypes. The majority of the STs found in the wild bird species have not been associated with human disease in the *Campylobacter jejuni* and *Campylobacter coli* MLST database. The most common were ST-1020, which was represented by 63 isolates (all from UK starlings), and ST-177, which was represented by 49 isolates mostly from UK starlings, but also from bird species in the genus *Turdus* (thrushes). A further 26 STs were represented by ten or more isolates in this data set (Table S1).

### Population structure among hosts

*Campylobacter jejuni* genotypes from the different wild bird host species were genetically distinct from each other, as well as from the genotypes typically recovered from humans and food animals. With nucleotide-based analyses, the values of genetic subdivision (*F*_ST_, which has possible values between 0 = no and 1 = complete subdivision) were on average 0.420 for the concatenated sequence from all loci (Table S2, Supporting information). The genetic differentiation among isolates from different host sources was well resolved in a neighbour-joining tree (Fig. 1). Isolates from food animals and humans clustered together and showed relatively little genetic subdivision (mean *F*_ST_ among these populations was 0.064, SD ± 0.025), while wild bird *C. jejuni* populations showed high genetic subdivisions and long branch lengths (Fig. 1). Notably, host species taxonomy rather than geographic origin was the main informative criterion for clustering, where populations of bacteria from taxonomically related bird host species had low levels of genetic differentiation, for example, black-headed gulls vs. silver gulls (*F*_ST_ = 0.019), and Dunlins vs. sharp-tailed sandpipers (*F*_ST_ = 0.051). Also, Mallards and geese had genetically similar *C. jejuni* populations (*F*_ST_ = 0.078; Table S2). In fact, for these three comparisons, the *F*_ST_ values were in similar ranges to those observed in the population sets of domestic animals and humans.

Population differentiation occurred also at the level of allele distribution, although the pattern was not as clear as for nucleotide polymorphisms (Fig. S1). Again, populations of *C. jejuni* from related wild bird species tended to cluster together, and humans and food animals were most closely related, an exception being chicken *C. jejuni* from Senegal that clustered away from the other farm animal subsets. However, the *F*_ST_ values were generally lower than the nucleotide-based values (overall *F*_ST_ = 0.070) and the resulting tree less resolved.

The ClonalFrame algorithm was used for genealogical reconstructions. This evolutionary-based approach reconstructed a genealogy with a topology consistent with the relationships deduced on the basis of population genetic subdivision, which is suitable for these types of recombinant bacteria. Generally, wild bird genotypes clustered away from farm animal–related genotypes, and wild bird species genotypes from taxonomically related species tended to cluster together (Fig. 2). A similar pattern was observed for clonal relationships in the entire data set, where the resulting minimum spanning tree showed clear separation of farm animal–associated genotypes and genotypes from wild bird hosts (Fig. S2).

Finally, when using structure analysis, most human isolates were assigned to farm animal origin (Fig. 3). Over 98% assignment of the 153 Australian isolates from humans was attributed to a farm animal source (cattle, sheep or chicken) and less than 2% to wild bird species. Just over 75% of UK isolates from humans were attributed to farm animals with 15% attributed to shorebirds, 7% to mallards and 1% each to gulls and geese.

### Temporal and geographical variation in genotypes

To assess the temporal stability of *C. jejuni* genotypes recovered from the different hosts in more detail, analysis of molecular variance (amova) were performed for isolates from wild bird species where data had been collected in different years, in different migration seasons within a year (spring vs. fall migration) or in different geographical places (Table S1). Due to trapping and sampling effects, as well as in the case of the song thrush where prevalence rates differed between seasons, only two of the major wild bird host species could be analysed for season and year within a country. In the first species, the analysis of black-headed gull *C. jejuni* incorporated the effects of sampling year and season within the year. Year or season accounted for only 0.7% (*F*_ST_ = 0.007, *P* < 0.001) and 0.9% (*F*_ST_ = 0.009, *P* = 0.37) of the variation, respectively, and nearly, all molecular variation was found within season within year (98.4%, *F*_ST_ = 0.016, *P* < 0.001). The analysis of isolates from Swedish blackbirds investigated the effect of season of the year (spring or autumn) and the sex of the birds (male or female). There were large differences among *C. jejuni* genotypes depending on whether they had been sampled during the spring or autumn migrations of the birds, accounting for 11% of the total molecular variance (*F*_ST_ = 0.118, *P* < 0.001). No effect of gender was seen, and the remaining variance was best explained by variation within the sexes within the seasons (*F*_ST_ = 0.116, *P* < 0.001).

Data from Swedish starlings and mallards were compared with the data from 50 starling and 50 goose isolates collected in Oxford, UK. These UK samples were randomly chosen from the larger collection of samples used in the genetic distance analyses and had been collected in three and two different years, respectively (Colles *et al*. 2008a, 2009). A structured amova on starlings, incorporating sampling country (Sweden and UK) and year of sampling, showed that there was hardly no effect of sampling country (*F*_ST_ = 0.022, *P* = 0.24), moderate effects of sampling year (6.3% of the variance explained, *F*_ST_ = 0.061 *P* < 0.001) and large effects within population within year (95.6% of the variance explained, *F*_ST_ = 0.040 *P* < 0.001). Similar patterns were seen in the comparison between UK geese and Swedish mallards; no effects of host species, which in this case also equals country of origin (*F*_ST_ = 0.002 *P* = 0.34), moderate year effects (explaining 13.0% of the molecular variance, *F*_ST_ = 0.130 *P* = 0.002) and large within population within year effects (87.2% of the variance, *F*_ST_ = 0.130 *P* < 0.001).

## Discussion

The *C. jejuni* populations investigated showed strong patterns of genetic subdivision, with distinct genetic subsets associated with particular hosts. The most genetically distinct *C. jejuni* populations were isolated from different wild bird species, with farm animal isolates much more similar to each other (Fig. 1). Furthermore, the genetic population structure was clearly associated with host taxonomy, with *C. jejuni* populations from taxonomically closely related bird species being most similar to each other (Fig. S2). The host associations were clearly evident in *F*_ST_ analyses, where values based on the nucleotide sequences between *C. jejuni* populations from some of the wild bird species were several times larger than the corresponding values between *C. jejuni* populations in different farm animal species (Table S1). A similar, but not as strong pattern was observed at the allele level (Fig. S1), and a phylogenetic consensus tree of randomly sampled genomes from a reduced set of source populations also supported the notion of strong separation of *C. jejuni* populations by wild bird host species and not by geographic location (Fig. 2).

Strong differentiation at the nucleotide level and weaker differentiation at the allele level suggests that the observed population genetic structure is fairly old and not the result of recent introduction to particular hosts. The overall sequence diversity was 3.1%, well within the subspecies range for *Campylobacter* spp., and only few alleles were unique to a specific wild bird host. The population structure of *C. jejuni* comprises multiple clonal complexes of related genotypes, which exhibit high rates of recombination, but with little deeper clonal structure evident among clonal complexes in the analysis of 7 locus mlst data (Dingle *et al*. 2001; Manning *et al*. 2001; Suerbaum *et al*. 2001). The data collected here suggest that host associations are important determinants of the genotypes isolated from nonfood animal sources, and raise the question whether the structure observed in food animals is the consequence of more recent introductions and expansions of certain genotypes in this novel niche. The limited host range for some of the genotypes obtained from wild birds, as well as the general genetic subdivision detected in these analyses, suggests restriction to gene flow dependent on host species or the existence of host-adapted strains of *C. jejuni*. Some of the sampled bird species co-occur in time and space in such a way that transmission of bacterial genotypes should be possible. For instance, blackbirds and song thrushes occupy slightly different ecological niches in forested areas in Europe, but occur, side by side, during migration and on wintering sites. Similarly, shorebirds and gulls (both in Europe and in Australia) feed intensively in the littoral zone, at times together in large aggregations. Some shared genotypes were seen between the two species of thrushes, as well as between gulls and shorebirds, but the general picture was that these hosts served as different bacterial niches. Most remarkably, among blackbirds sampled in Australia, two of the four detected genotypes (ST-1324 and ST-1342) were shared with blackbirds sampled in Sweden, and the remaining two (ST-3067 and -3068) were very similar. We do not know whether the blackbird-associated genotypes occur in other Australian birds, or whether they are restricted to this single host species. If the former case is true, then the shared polymorphisms in *C. jejuni* strains occurring in an allopatric single host species strongly indicate host species adaptations, especially because the Australian blackbirds date back to the European colonization of Australia in the 19th century (Higgins *et al*. 2006).

Most studies that have compared serotypes or genotypes from wild birds with poultry and patient isolates have typically identified predominantly unique strains of *C. jejuni* in wild birds, for example, in herring gulls *Larus argentatus* from Scotland (Whelan *et al*. 1988), feral pigeons from Japan (Fukuyama *et al*. 1986), various wild bird species in Norway (Rosef *et al*. 1985) and starlings in the UK (Colles *et al*. 2003, 2009), indicating that wild birds are not a significant direct source of human campylobacteriosis. Recently, Sheppard and co-workers performed attribution analyses on *C. jejuni* strains from food and wild animal sources restricted to the United Kingdom and suggested strong niche associations in genotypes from wild birds with shared feeding ecology, including ducks and geese, and pigeons and doves (Sheppard *et al*. 2011). The data presented here confirm the hypothesis of host association in wild bird *C. jejuni* populations and provide evidence that this occurs across large spatial and temporal scales. The level of host associations seems to differ depending on both *C. jejuni* strain and host species, for instance in mallard and geese that showed shared *C. jejuni* populations despite being taxonomically less related than the other wild bird pairs investigated.

Host associations that transcend geographic boundaries have been shown in a global collection of *Pasteurella multocida* from ovine, avian, porcine and bovine sources (Hotchkiss *et al*. 2011). For this pathogen, a niche association was also identified, similar to our study. Furthermore, in *Staphylococcus aureus*, clonal complexes are sometimes associated with a specific source, such as humans, cattle or other ruminants, across several continents (Smyth *et al*. 2009).

Some of the STs found in wild birds have been found in food animals or in human patients, including representatives from the widespread ST-21, ST-45 and ST-283 clonal complexes. In some of these cases, the wild birds may have acquired food animal–associated *C. jejuni* genotypes through proximity to these sources. For instance, black-headed gulls are an opportunistic feeder, and not uncommon in agricultural or urban areas. Likewise, in Europe, a proportion of European song thrushes and blackbirds breed and/or winter in gardens and parks and are thereby also potentially exposed to human and farm animal–related *C. jejuni* genotypes. In this perspective, it is noteworthy that human disease-associated STs were only isolated from blackbirds during spring migration when birds returned from wintering grounds in continental Europe and in the UK to Sweden.

The attribution analysis (Fig. 3) indicated that the majority of isolates from human campylobacteriosis have STs indicative of a farm animal origin. This was also true for Australian isolates despite the fact that the farm animal isolates used for attribution originated from the UK and West Africa, whereas some of the wild bird isolates were Australian. Although farm animal subtypes have been suggested to be more dependent on host specificity than geography (Sheppard *et al*. 2010, 2011), the level of such host associations is clearly less pronounced compared to that observed in wild birds, and there are frequent examples of genotypes in farm animal studies that were not associated with a single host species.

In conclusion, the *C. jejuni* genotypes isolated from wild bird hosts exhibited substantially different population structure from that seen in isolates from food animal sources, as suggested also by previous studies (Broman *et al*. 2004; Colles *et al*. 2008a; Sheppard *et al*. 2011). Importantly, there was also a relative lack of secular temporal and phylogeographic effects, suggesting that the differentiation by host is old relative to current genotype distribution and that host species–genotype associations are robust in wild bird *C. jejuni*. A zoonotic, multiple host bacterial pathogen, such as *C. jejuni*, has many different selection forces operating on its epidemiology. Not only has it to evolve mechanisms to persist and multiply in different enteric environments, which could differ in temperature, structure and biochemical and immunological habitats, but it also has to evolve means to successfully survive in environments during transmission. In such a setting, two survival strategies can be postulated (i) adaptations favouring the colonization and persistence in one certain host species; or (ii) adaptations favouring transmission and colonization of several hosts. There is evidence for both these strategies in *C. jejuni* (i) genotypes that are widespread among hosts (multihost lineages), for example, the ST-45 complex and several other food animal–associated genotypes; and (ii) genotypes that are confined to single hosts (host specific lineages) such as the ST-1264 and ST-1347 complexes found in song thrushes. Strain-specific properties in colonization abilities have been noted previously in chickens, both in terms of host (Stern *et al*. 1990) and of bacterial genotypes (Stern *et al*. 1988; Cawthraw *et al*. 1996; Payne *et al*. 1999), and similarly, strain-specific differences in survival times in water environments have been observed (Obiri-Danso *et al*. 2001; Cools *et al*. 2003). It is interesting to note that multihost genotypes dominate in food animals and in human infections (Sheppard *et al*. 2011), suggesting that a broad host range is associated with the emergence of human campylobacteriosis. This study thus demonstrates that *C. jejuni* isolated from wild birds are in general distinct from those isolated from human campylobacteriosis and food animals. Further, there is strong differentiation by wild bird host species, which may be the biologically important niche for this bacterium. The population diversity for this niche described here, when compared to that found in *C. jejuni* isolated from food animals, demonstrates the small portion of the whole, which is visible from the anthropocentric view of the ecology of *C. jejuni*.

## Notes

P.G., F.C., N.M., P.H., C.A-S., B.O., D.H., M.M. and J.W. designed research. P.G., F.C., P.H., C.A-S., B.O., and J.W. collected data. P.G., F.C., N.M., B.O., and J.W. analysed data. P.G., F.C., N.M., P.H., C.A-S., B.O., D.H., M.M. and J.W. wrote the paper. The authors declare no conflict of interest.

## Data accessibility

Data on Swedish and Australian wild bird *C. jejuni* isolates have been deposited in the *Campylobacter jejuni/coli* PubMLST database (http://pubmlst.org/campylobacter/) with accession numbers 21601-22077, and an alignment can be downloaded at Dryad (doi:10.5061/dryad.8p2f7). Other analysed sequence data were gathered from published studies on *C. jejuni* in humans and farm animals (Kinana *et al*. 2006; McCarthy *et al*. 2007; Mickan *et al*. 2007; Dingle et al. 2008).

## Supporting information

**Fig. S1** Unrooted neighbor-joining tree displaying the pairwise genetic distances (*F*_ST_ values) between *Campylobacter jejuni* populations from different hosts and geographical areas. The *F*_ST_ values were calculated from observed MLST allele distributions in the 2294 *C. jejuni* isolates

**Fig. S2** (A) Full Minimum Spanning Tree of 2294 *Campylobacter jejuni* isolates typed with MLST. The size of each circle is proportional (on a logarithmic scale) to the number of isolates with that particular ST in the total sample. STs are indicated by the number in each circle. Black lines connect STs that differ from each other at a single locus, while dark grey and light grey lines connect STs differing at two and three loci, respectively. The proposed founders of particular clusters are indicated by a light green outer circle. The subfounders (defined has having links to three or more STs) are indicated by dark green outer circles. Human and food animal related isolates are shown in grey while the isolates of different wild birds are denoted in colours: silver gull (light green), black-headed gull (dark green), blackbird (red), song thrush (purple), European starling (pink), goose species (yellow), mallards (orange), sharp-tailed sandpiper (dark blue), dunlin (light blue). For visualisation, the parts of the tree containing wild bird samples have been enlarged (B, C and D).

**Table S1** Occurrence of different STs among sampled *Campylobacter jejuni* populations, sorted by clonal complex. Wild bird *C. jejuni* isolate data is deposited in the *C. jejuni/coli* PubMLST database (http://pubmlst.org/campylobacter/) with accession numbers 21601-22077.

**Table S2** Pair-wise genetic distance data among 17 subpopulations of *Campylobacter jejuni*. The upper-right section show *F*_ST_ values based on MLST allele distributions and the lower-left shows *F*_ST_ values based on nucleotide polymorphisms
