# Supplementary material for: Marked host specificity and lack of phylogeographic population structure of Campylobacter jejuni in wild birds
Source: Mol Ecol. 2013 Jan 29;22(5):1463–72. doi: 10.1111/mec.12144 (PMC3596980; doi:10.1111/mec.12144)
Supplement: Supplementary file 3 [file mec0022-1463-SD3.pdf]

Table S1. Occurrence of different STs among sampled *C. jejuni* populations, sorted by clonal complex. Wild bird *C. jejuni* isolate data is deposited in the Campylobacter jejuni/coli PubMLST database (http://pubmlst.org/campylobacter/) with accession numbers 21601-22077.

Clonal complex	ST	Blackbird Australia	Blackbird Sweden	Starling UK	Starling Sweden	Sharp-tailed Sandpiper Australia	Dunlin Sweden	Silver Gull Australia	Black-headed Gull Sweden	Mallard Sweden	Song Thrush Sweden	Goose sp. UK	Human UK	Human Australia	Total
ST-21 complex	19												15		15
ST-21 complex	21												29	2	31
ST-21 complex	44												3		3
ST-21 complex	47												2		2
ST-21 complex	50							2	1				24	7	34
ST-21 complex	53												16	1	17
ST-21 complex	104												13		13
ST-21 complex	141												1		1
ST-21 complex	148												1		1
ST-21 complex	169												1		1
ST-21 complex	186												1		1
ST-21 complex	190													1	1
ST-21 complex	193											1			1
ST-21 complex	262												5		5
ST-21 complex	266												1		1
ST-21 complex	536													1	1

ST-21 complex	569						1	1
ST-21 complex	806					1		1
ST-21 complex	822					2		2
ST-21 complex	1383		1					1
ST-22 complex	22					6		6
ST-22 complex	567						1	1
ST-42 complex	42		1			5	4	10
ST-42 complex	447					3		3
ST-42 complex	451						2	2
ST-45 complex	11	5						5
ST-45 complex	25						2	2
ST-45 complex	45	1	5	3		23	1	33
ST-45 complex	137	1			7	6		14
ST-45 complex	233					1	1	2
ST-45 complex	334		1					1
ST-45 complex	418					1		1
ST-45 complex	529						1	1
ST-45 complex	538			1			1	2
ST-45 complex	583					5		5
ST-45 complex	706				1			1
ST-45 complex	998		1					1
ST-45 complex	1003			1				1
ST-45 complex	1025		1					1
ST-45 complex	1326			1				1
ST-48 complex	38		2					2
ST-48 complex	48					19	23	42
ST-48 complex	66					1		1
ST-48 complex	205					1		1
ST-48 complex	475					7		7
ST-49 complex	49					6		6
ST-52 complex	52					2	5	7
ST-52 complex	70						1	1
ST-52 complex	161						4	4
ST-52 complex	533						1	1

ST-52 complex	775				1		1
ST-61 complex	61				10	1	11
ST-61 complex	93				1		1
ST-61 complex	568					1	1
ST-177 complex	144		3				3
ST-177 complex	177	1	46	2			49
ST-177 complex	563		2				2
ST-177 complex	685		1				1
ST-177 complex	1004		3				3
ST-177 complex	1014		1				1
ST-177 complex	1329			1			1
ST-177 complex	1381		2				2
ST-177 complex	1382		2				2
ST-177 complex	1388		2				2
ST-177 complex	1394		3				3
ST-177 complex	1482		1				1
ST-177 complex	1485		1				1
ST-177 complex	1500		1				1
ST-177 complex	1506		1				1
ST-177 complex	1533		1				1
ST-177 complex	1535		1				1
ST-179 complex	220		3				3
ST-206 complex	46				1		1
ST-206 complex	206				10		10
ST-206 complex	227					5	5
ST-206 complex	227				3		3
ST-206 complex	273				1		1
ST-206 complex	572				8		8
ST-257 complex	197					2	2
ST-257 complex	257		2		47	18	67
ST-257 complex	532					2	2
ST-257 complex	584				1		1
ST-257 complex	824				4		4
ST-257 complex	1380				1		1

ST-283 complex	267	1	1	5		7
ST-353 complex	5			15	3	18
ST-353 complex	82			1		1
ST-353 complex	353			4		4
ST-353 complex	356			1		1
ST-353 complex	400			7		7
ST-353 complex	524				2	2
ST-353 complex	527				2	2
ST-353 complex	537				1	1
ST-353 complex	581			2		2
ST-354 complex	354			11	3	14
ST-354 complex	528				18	18
ST-362 complex	27			1		1
ST-403 complex	270			1		1
ST-403 complex	403			1		1
ST-443 complex	51			37	1	38
ST-443 complex	443			1		1
ST-443 complex	1396			1		1
ST-446 complex	446			1		1
ST-446 complex	450			2		2
ST-460 complex	460			1		1
ST-460 complex	535				1	1
ST-460 complex	606			1		1
ST-464 complex	364			1		1
ST-464 complex	464			3		3
ST-508 complex	508			2		2
ST-573 complex	325			1		1
ST-573 complex	573			4		4
ST-574 complex	574		1	27		28
ST-574 complex	1599			1		1
ST-607 complex	525				6	6
ST-607 complex	607			4		4
ST-658 complex	312			1	1	2
ST-658 complex	523			2	7	9

ST-658 complex	658									9	9
ST-658 complex	1395									1	1
ST-658 complex	1398									1	1
ST-661 complex	814									1	1
ST-677 complex	677		4	2				1			7
ST-677 complex	1024			1							1
ST-677 complex	1534			1							1
ST-682 complex	681			1							1
ST-682 complex	682			9	4						13
ST-682 complex	686			15							15
ST-682 complex	687			1							1
ST-682 complex	818			11							11
ST-682 complex	1019			1							1
ST-682 complex	1020			63							63
ST-682 complex	1021			4							4
ST-682 complex	1022			13							13
ST-682 complex	1027			13							13
ST-682 complex	1385			1							1
ST-682 complex	1386			1							1
ST-682 complex	1387			1							1
ST-682 complex	1390			1							1
ST-682 complex	1391			1							1
ST-682 complex	1392			1							1
ST-682 complex	1503			2							2
ST-682 complex	1505			1							1
ST-682 complex	1507			1							1
ST-682 complex	1542			2							2
ST-682 complex	3068	1									1
ST-692 complex	692						5	1	4		10
ST-692 complex	699					1	1		7		9
ST-692 complex	707								6		6
ST-692 complex	991						13				13
ST-692 complex	1278						2				2
ST-692 complex	1280						1				1

ST-692 complex	1301		1					1
ST-692 complex	1339			2				2
ST-702 complex	702					18		18
ST-702 complex	703					1		1
ST-702 complex	705					1		1
ST-702 complex	1333			1				1
ST-702 complex	1350			1				1
ST-828 complex	825						5	5
ST-828 complex	826						1	1
ST-828 complex	827						10	10
ST-952 complex	1310		1					1
ST-1034 complex	694					2		2
ST-1034 complex	697					1		1
ST-1034 complex	698					1		1
ST-1034 complex	711			1		2		3
ST-1034 complex	788					1		1
ST-1034 complex	977		3			1		4
ST-1034 complex	1029					1		1
ST-1034 complex	1033			1		16		17
ST-1034 complex	1034			1				1
ST-1034 complex	1255	1		13				14
ST-1034 complex	1269		1	2				3
ST-1034 complex	1299			1				1
ST-1034 complex	1327			1				1
ST-1034 complex	1335		1					1
ST-1034 complex	1354		1					1
ST-1034 complex	1608					1		1
ST-1264 complex	1250				1			1
ST-1264 complex	1259				12			12
ST-1264 complex	1264				24			24
ST-1264 complex	1293				1			1
ST-1264 complex	1320				1			1
ST-1264 complex	1353				2			2
ST-1264 complex	1356				2			2

ST-1275 complex	637			4	8	12
ST-1275 complex	1223			2	28	30
ST-1275 complex	1225				3	3
ST-1275 complex	1268				5	5
ST-1275 complex	1274				4	4
ST-1275 complex	1275				5	5
ST-1275 complex	1283				2	2
ST-1275 complex	1292				6	6
ST-1275 complex	1298				1	1
ST-1275 complex	1330				1	1
ST-1275 complex	1334				1	1
ST-1275 complex	1336				1	1
ST-1275 complex	1341				2	2
ST-1275 complex	3047			1		1
ST-1275 complex	3048			1		1
ST-1275 complex	3049			2		2
ST-1275 complex	3051			1		1
ST-1275 complex	3060			1		1
ST-1275 complex	3061			1		1
ST-1275 complex	3063			1		1
ST-1275 complex	3064			1		1
ST-1287 complex	1253	3	2			5
ST-1287 complex	1287	1	2		2	5
ST-1287 complex	1288		3			3
ST-1287 complex	1305		1			1
ST-1287 complex	1312		1			1
ST-1287 complex	1317	3				3
ST-1287 complex	1328	1	1			2
ST-1287 complex	1345		5			5
ST-1287 complex	1346		1			1
ST-1287 complex	1352	5	5			10
ST-1287 complex	1355		2			2
ST-1287 complex	3037	6		1		7
ST-1287 complex	3039	5				5

ST-1287 complex	3040			3							3
ST-1287 complex	3042			1							1
ST-1287 complex	3046			1							1
ST-1287 complex	3052			1							1
ST-1287 complex	3053			2							2
ST-1287 complex	3054			1							1
ST-1287 complex	3056			1							1
ST-1287 complex	3059			1							1
ST-1304 complex	1304	5					5				10
ST-1304 complex	1307						1				1
ST-1304 complex	1315						1				1
ST-1304 complex	1369						1				1
ST-1325 complex	1271	1									1
ST-1325 complex	1325	2					1				3
ST-1325 complex	1331	1									1
ST-1332 complex	696					2		9			11
ST-1332 complex	994					1					1
ST-1332 complex	1276					4					4
ST-1332 complex	1332				1	3					4
ST-1347 complex	1291						1				1
ST-1347 complex	1311						1				1
ST-1347 complex	1344						1				1
ST-1347 complex	1347						14				14
ST-1347 complex	1368						1				1
Unassigned	350								1		1
Unassigned	436		2						3		5
Unassigned	441								1		1
Unassigned	449									1	1
Unassigned	466								2		2
Unassigned	526									1	1
Unassigned	530									7	7
Unassigned	531								1	10	11
Unassigned	586								1		1
Unassigned	683		14								14

Unassigned	684		1					1
Unassigned	691					5		5
Unassigned	693				4	9		13
Unassigned	695					5		5
Unassigned	700			1		8		9
Unassigned	701					8		8
Unassigned	704				2	3		5
Unassigned	708					1		1
Unassigned	709					6		6
Unassigned	710					13		13
Unassigned	785					1		1
Unassigned	786					1		1
Unassigned	789					1		1
Unassigned	801						1	1
Unassigned	821						1	1
Unassigned	992				3			3
Unassigned	993				2	1		3
Unassigned	995				2			2
Unassigned	996			2	7			9
Unassigned	997		2					2
Unassigned	999		1					1
Unassigned	1000		1					1
Unassigned	1002		1					1
Unassigned	1023		9					9
Unassigned	1026		1					1
Unassigned	1028					3		3
Unassigned	1030				1	4	1	6
Unassigned	1031					10		10
Unassigned	1032					2		2
Unassigned	1080	1					1	2
Unassigned	1229	8						8
Unassigned	1249	2						2
Unassigned	1252	1						1
Unassigned	1254	1						1

Unassigned	1256					1			1
Unassigned	1257						1		1
Unassigned	1260		9						9
Unassigned	1261					1			1
Unassigned	1263						1		1
Unassigned	1265						1		1
Unassigned	1266		6						6
Unassigned	1267						1		1
Unassigned	1277		4						4
Unassigned	1279					1			1
Unassigned	1285					1			1
Unassigned	1286		3						3
Unassigned	1289					2			2
Unassigned	1290		3						3
Unassigned	1294						2		2
Unassigned	1296							1	1
Unassigned	1297		1						1
Unassigned	1303		10						10
Unassigned	1308						2		2
Unassigned	1313				1				1
Unassigned	1314				2				2
Unassigned	1316		6						6
Unassigned	1318							6	6
Unassigned	1321				2				2
Unassigned	1322					2			2
Unassigned	1323							4	4
Unassigned	1324	1	5						6
Unassigned	1337		1						1
Unassigned	1342	1	12					1	14
Unassigned	1343					1			1
Unassigned	1351		3						3
Unassigned	1367		5						5
Unassigned	1384			1					1
Unassigned	1389			1					1

Unassigned	1397												1		1
Unassigned	1481			1											1
Unassigned	1483			1											1
Unassigned	1484			1											1
Unassigned	1486			1											1
Unassigned	1501			1											1
Unassigned	1502			1											1
Unassigned	1504			1											1
Unassigned	1508			1											1
Unassigned	1536			1											1
Unassigned	1537			1											1
Unassigned	1539			1											1
Unassigned	1606											3			3
Unassigned	1607											1			1
Unassigned	1609			1											1
Unassigned	1612			1											1
Unassigned	1613			1											1
Unassigned	3041					1									1
Unassigned	3043					1									1
Unassigned	3044					1									1
Unassigned	3045					1									1
Unassigned	3050					3									3
Unassigned	3055					2									2
Unassigned	3057					1									1
Unassigned	3062							1							1
Unassigned	3065							1							1
Unassigned	3066							1							1
Unassigned	3067	1													1
Unassigned	3070					1									1
		4	103	285	7	46	29	22	97	85	84	166	470	153	1551
