# Supplementary material for: Marked host specificity and lack of phylogeographic population structure of Campylobacter jejuni in wild birds
Source: Mol Ecol. 2013 Jan 29;22(5):1463–72. doi: 10.1111/mec.12144 (PMC3596980; doi:10.1111/mec.12144)
Supplement: Supplementary file 5 [file mec0022-1463-SD5.doc]

TABLE S2. Pair-wise genetic distance data among 17 subpopulations of *C. jejuni*. The upper-right section show FST values based on MLST allele distributions and the lower-left shows FST values based on nucleotide polymorphisms

| Population | [1] | [2] | [3] | [4] | [5] | [6] | [7] | [8] | [9] | [10] | [11] | [12] | [13] | [14] | [15] | [16] | [17] |
| --- | --- | --- | --- | --- | --- | --- | --- | --- | --- | --- | --- | --- | --- | --- | --- | --- | --- |
| [1] Black-headed Gulls Sweden |  | 0.064 | 0.075 | 0.073 | 0.102 | 0.053 | 0.084 | 0.071 | 0.080 | 0.058 | 0.078 | 0.074 | 0.075 | 0.030 | 0.116 | 0.184 | 0.092 |
| [2] Blackbirds Australia | 0.367 |  | 0.010 | 0.032 | 0.067 | 0.010 | 0.043 | 0.030 | 0.040 | 0.016 | 0.038 | 0.030 | 0.033 | 0.024 | 0.084 | 0.193 | 0.056 |
| [3] Blackbirds Sweden | 0.409 | 0.051 |  | 0.050 | 0.077 | 0.032 | 0.059 | 0.048 | 0.057 | 0.037 | 0.055 | 0.049 | 0.051 | 0.045 | 0.087 | 0.154 | 0.068 |
| [4] Cattle UK | 0.363 | 0.310 | 0.381 |  | 0.076 | 0.020 | 0.059 | 0.049 | 0.048 | 0.022 | 0.055 | 0.049 | 0.006 | 0.045 | 0.090 | 0.155 | 0.068 |
| [5] Chicken UK | 0.311 | 0.290 | 0.355 | 0.070 |  | 0.058 | 0.087 | 0.074 | 0.080 | 0.061 | 0.081 | 0.076 | 0.075 | 0.073 | 0.120 | 0.191 | 0.095 |
| [6] Chickens Senegal | 0.431 | 0.366 | 0.444 | 0.098 | 0.095 |  | 0.041 | 0.031 | 0.031 | 0.007 | 0.038 | 0.031 | 0.023 | 0.022 | 0.073 | 0.135 | 0.050 |
| [7] Dunlins Sweden | 0.695 | 0.571 | 0.656 | 0.551 | 0.591 | 0.673 |  | 0.057 | 0.066 | 0.045 | 0.059 | 0.033 | 0.060 | 0.054 | 0.104 | 0.174 | 0.079 |
| [8] Geese UK | 0.481 | 0.410 | 0.479 | 0.263 | 0.278 | 0.307 | 0.605 |  | 0.055 | 0.035 | 0.046 | 0.047 | 0.049 | 0.043 | 0.088 | 0.153 | 0.067 |
| [9] Humans Australia | 0.384 | 0.331 | 0.416 | 0.073 | 0.070 | 0.089 | 0.609 | 0.297 |  | 0.024 | 0.061 | 0.055 | 0.048 | 0.048 | 0.097 | 0.162 | 0.074 |
| [10] Humans UK | 0.254 | 0.231 | 0.288 | 0.039 | 0.042 | 0.068 | 0.430 | 0.194 | 0.045 |  | 0.042 | 0.036 | 0.023 | 0.028 | 0.076 | 0.139 | 0.054 |
| [11] Mallards Sweden | 0.504 | 0.420 | 0.501 | 0.286 | 0.314 | 0.354 | 0.533 | 0.078 | 0.307 | 0.212 |  | 0.054 | 0.056 | 0.049 | 0.096 | 0.162 | 0.074 |
| [12] Sharp-tailed Sandpipers Australia | 0.658 | 0.549 | 0.630 | 0.532 | 0.566 | 0.646 | 0.051 | 0.590 | 0.583 | 0.414 | 0.524 |  | 0.050 | 0.038 | 0.092 | 0.158 | 0.068 |
| [13] Sheep UK | 0.363 | 0.307 | 0.378 | 0.006 | 0.067 | 0.077 | 0.564 | 0.265 | 0.080 | 0.038 | 0.297 | 0.544 |  | 0.045 | 0.092 | 0.156 | 0.069 |
| [14] Silver Gulls Australia | 0.019 | 0.312 | 0.372 | 0.281 | 0.239 | 0.332 | 0.597 | 0.388 | 0.297 | 0.194 | 0.407 | 0.562 | 0.276 |  | 0.090 | 0.161 | 0.065 |
| [15] Song Thrushes Sweden | 0.586 | 0.274 | 0.300 | 0.526 | 0.534 | 0.603 | 0.783 | 0.618 | 0.586 | 0.420 | 0.637 | 0.759 | 0.521 | 0.544 |  | 0.205 | 0.109 |
| [16] Starlings Sweden | 0.689 | 0.488 | 0.627 | 0.598 | 0.615 | 0.726 | 0.894 | 0.734 | 0.658 | 0.457 | 0.741 | 0.867 | 0.604 | 0.635 | 0.786 |  | 0.124 |
| [17] Starlings UK | 0.531 | 0.344 | 0.485 | 0.462 | 0.453 | 0.561 | 0.753 | 0.595 | 0.499 | 0.346 | 0.602 | 0.727 | 0.465 | 0.484 | 0.656 | 0.127 |  |
